# Adjunct Non-Elastic Hip Taping Improves Gait Stability in Cane-Assisted Individuals with Chronic Stroke: A Randomized Controlled Trial

**DOI:** 10.3390/jcm11061553

**Published:** 2022-03-11

**Authors:** Ray-Yau Wang, Chieh-Yu Lin, Jyue-Liang Chen, Chun-Shou Lee, Yun-Ju Chen, Yea-Ru Yang

**Affiliations:** 1 Department of Physical Therapy and Assistive Technology, National Yang Ming Chiao Tung University, Taipei 11221, Taiwan; rywang@nycu.edu.tw; 2 Department of Physical Therapy and Assistive Technology, National Yang-Ming University, Taipei 11221, Taiwan; 3 Department of Physical Medicine and Rehabilitation, Taipei Veterans General Hospital, Taipei 11217, Taiwan; chiehyu516@gmail.com; 4 Department of Rehabilitation Medicine, Taipei Tzu Chi Hospital, Buddhist Tzu Chi Medical Foundation, New Taipei City 23142, Taiwan; c96420@gmail.com; 5 Section of Physical Therapy, Department of Rehabilitation Medicine, Renai Branch of Taipei City Hospital, Taipei 10629, Taiwan; a0019@tpech.gov.tw; 6 Section of Physical Therapy, Department of Rehabilitation Medicine, Zhongxing Branch of Taipei City Hospital, Taipei 10341, Taiwan; yunjuc@hotmail.com

**Keywords:** non-elastic tape, gait stability, cardiovascular disease, chronic stroke, rehabilitation

## Abstract

Cane-assisted individuals with chronic stroke may perform with an abnormal gait pattern. One of the important factors of gait training for cane-assisted individuals is inducing improvement in lower limb muscle activity of the paretic side. Non-elastic taping on the hip may be used as an adjunct therapy for improving gait. The objective of this study was to investigate effects of non-elastic hip taping combined with exercise on gait in cane-assisted individuals with chronic stroke. This study is a single-blinded, randomized controlled trial. A total of 21 cane-assisted ambulators with chronic stroke were enrolled. Participants in both groups received a therapeutic exercise program, with the experimental and control groups having adjunct non-elastic taping and sham taping on the hip, respectively. The gait, Berg Balance Scale, 6-min walk test, and Fall Efficacy Scale–International were measured at pre-intervention, post-intervention, and 1-month follow-up. The experimental group resulted in significantly better performance in double-support time compared with the control group. Furthermore, the experimental group showed a significant improvement in double-support time and spatial symmetry at post-intervention and 1-month follow-up compared with pre-intervention. This study demonstrated that non-elastic hip taping combined with exercise could improve gait stability in cane-assisted ambulators. Non-elastic hip taping would be a useful adjunct to rehabilitation strategies for individuals with chronic stroke.

## 1. Introduction

Stroke is a major cause of disability worldwide [1]. Physical disabilities after stroke usually lead to difficulties in moving, walking, and performing activities of daily living. Disability after stroke usually generates a chronic disease that requires continuous evaluation and assistance, even many years from the onset of the stroke [2,3]. Regaining the ability to walk independently is a major focus in stroke rehabilitation [4,5]. A cane is often prescribed for post-stroke individuals with unstable gait. After unloading the stress of the weight-bearing tissue and increasing the base of support with a cane, gait stability and walking efficiency may be temporarily improved [6,7]. Also, a cane can decrease the fear of falling and increase walking confidence via increasing the base of support in those with strokes. However, a cane would reduce muscle activation of the anti-gravity muscles on the affected side during walking in individuals with stroke [8]. Previous studies have shown that cane-assisted, post-stroke ambulators had poor balance and gait performance and less social participation than those who were independent of walking aids [9,10]. Sorensen et al. reported that, at a 3–5-year follow-up of post-stroke survivors with a walking aid, almost all of the survivors were still dependent on their walking aids [11]. Another study reported that long-term use of a cane may lead to negative effects on functional recovery due to the fact that the assistance of a cane causes joint and muscle unloading and decoupling from the central pattern generator’s control, which may adversely affect post-stroke plasticity and functional recovery [12]. Therefore, an effective intervention to rebuild gait stability and eliminate the need of walking aids is an important issue in stroke rehabilitation.

Exercise programs, including aerobic, strengthening, endurance, flexibility, and neuromuscular (balance, coordination) training, have been recommended to improve gait performance for individuals with stroke [13,14,15]. Several studies showed positive effects of aerobic, strengthening, and balance training on walking performance in individuals with stroke [13,16,17]. Motor imagery has also been shown to have a positive effect on the gait performance of individuals after stroke [18,19]. There are some studies focusing on the training effects for the cane-assisted, post-stroke ambulators. Previous studies showed that walking training with a weight support feedback cane leads to the improvement of gait ability in cane-assisted, post-stroke ambulators [20,21]. The most important factor of cane gait training is the progressive decrease in weight support on the cane. Such a training protocol might induce improvement in the lower limb muscle activity of the paretic side and gait performance [20,21]. Nevertheless, it is difficult to quantitatively measure weight support on the cane during gait training in a clinical setting. It is necessary to have alternative strategies to induce normal muscle activation of the lower limb on the paretic side.

Non-elastic taping is often applied to correct joint alignment and enhance muscle activity for generating force after musculoskeletal injuries [22,23]. The taping may place the muscle at a more mechanically advantageous length to enhance its contraction. Previous studies demonstrated the immediate effect of the gluteal taping and hip abductor taping on increasing the hip extension during the stance phase and increasing the hemiplegic hip abductors’ activity, respectively, in individuals with stroke [24,25]. Our recent study showed that non-elastic hip taping immediately improved balance, gait speed, and endurance in cane-assisted ambulators with chronic stroke [26]. Taken together, hip taping may be used to correct the pelvic position and activate the hip musculature and then to improve balance and gait. Therefore, it would be reasonable to infer that non-elastic hip taping could be as an adjunct therapy to exercise training in cane-assisted, post-stroke ambulators. The purpose of the present study was to investigate the effect of the non-elastic hip taping combined with exercise training on gait performance in cane-assisted ambulators with chronic stroke.

## 2. Materials and Methods

### 2.1. Ethical Procedure

The trial protocol was approved by the Institutional Review Board of Taipei City Hospital and was registered with the Australia New Zealand Clinical Trials Registry (ACTRN 12617000483358). The study was performed according to the ethical standards laid down in the Declaration of Helsinki. Prior to data collection, the purpose and procedure were fully explained, and informed consents were obtained from the participants.

### 2.2. Study Design

This study was a participant-blinded, randomized controlled trial. Assignment was performed by an independent person who selected one of a set of sealed envelopes before the intervention began. Participants were allocated randomly into either the experimental group or the control group. Participants in the experimental group and the control group received non-elastic taping and sham taping, respectively. After taping, participants underwent exercise training, including progressive resistance exercise, balance training, and treadmill training for 50 min per session, twice per week, for 6 weeks. Outcomes, including gait, balance, endurance, and concern about falling, were measured without any taping applied before the intervention, after the intervention, and at 1-month follow-up.

### 2.3. Participants

Participants were recruited from Taipei City Hospital in Taipei. Information on age, gender, height, weight, hemiparetic side, time since stroke, and duration of cane use were obtained through patient interviews and from medical records. All participants met the following inclusion criteria: (1) ischemic or hemorrhage stroke longer than 6 months, (2) walking with cane assistance, (3) a score of ≥3 in the functional ambulation category, (4) age between 20 and 80 years old, and (5) a score of ≥24 on the mini-mental state examination. The exclusion criteria were as follows: (1) a known allergy to adhesive sports tape, (2) a score of ≥2 on the Modified Ashworth Scale of the affected plantar flexors, (3) histories of other neurological, cardiovascular, or orthopedic diseases affecting walking ability, (4) histories of gross visuospatial or visual field deficits, and (5) inability to follow simple verbal instructions.

The effect size was estimated to be 1.21 from our previous study [26]. The sample size was determined using G*power based on the effect size of 1.21, an alpha level of 5%, 80% power, and a *t* test model. A minimum sample size of 20 participants was indicated. A total of 21 participants with chronic stroke was recruited and allocated into the experimental and control groups.

### 2.4. Intervention

Participants were requested to stand on the affected leg while placing the foot of the other leg on a step. Gluteal and hip abductor taping was applied on the affected side. The taping technique was the same as that in our previous study [26]. In brief, gluteal taping was applied first, and hip abductor taping was applied subsequently. Participants in the experimental group received both hypoallergenic tape and leukotape (real tape). First, hypoallergenic tape was applied without tension to protect the skin. Leukotape was then applied on the hip extensors prior to the hip abductors. Participants in the control group received hypoallergenic tape only (sham tape) on both the hip extensors and abductors. The tape was removed immediately after exercise training.

Participants in both groups performed exercise training with real tape or sham tape. The intervention program consisted of a 5 min warm up with light walking and stretching exercise, 15 min of progressive resistance training, 15 min of balance training, 10 min of treadmill training, and a 5 min cool down. The key muscles of progressive resistance training were hip extensors and abductors. An elastic resistance band was used for resistance training. The intensity of the exercise progressed from 10 to 15 repetitions with 2 to 3 sets for each exercise. The resistance level of the elastic band was gradually increased as tolerance improved over 6 weeks and was regulated with the assistance of ratings from 12 to 13 of the Borg rating of perceived exertion scale. Balance training included static and dynamic balance. Static balance training was carried out in standing position and was composed of functional tasks, such as reaching to markers, picking up an object from floor, catching and throwing a ball, etc. Dynamic balance training involved stepping forward/backward onto a step, obstacle crossing, tandem walking, sideways walking, braided walking, backward walking, and so on.

### 2.5. Outcome Measures

Outcome measures were administrated in the condition without a cane before the intervention, after the intervention, and at 1-month follow-up. The primary outcome was gait performance. The secondary outcomes were balance, endurance, and concern about falling.

Gait was measured with the OPTOgait system (Optojump Next, Microgate, Bolzano, Italy). The OPTOgait system consists of a corridor of light-emitting and light-receiving diodes that are placed parallel to each other and oriented perpendicular to the line of progression. The validity and test–retest reliability of gait measurement has been well established [27,28]. Participants were instructed to walk at a comfortable pace for three trials. Data were averaged from the three trials. The interest parameters were velocity, double-support time, spatial and temporal symmetry index. Step length and stance duration were used to calculate spatial and temporal symmetry index, respectively. The formula is that of symmetry index = |X_Left_ − X_Right_|/0.5 (X_Left_ + X_Right_) × 100%) [29].

Balance was evaluated with the Berg Balance Scale (BBS). The BBS is a 14-item scale that quantitatively assesses balance. The items are scored from 0 to 4, with a score of 0 representing an inability to complete the task, and a score of 4 representing independent item completion. A global score is calculated out of 56 possible points. The BBS is a valid and highly reliable tool for evaluating balance in the stroke population [30,31].

The 6-min walk test (6MWT) was used to evaluate walking endurance. Participants were instructed to walk back and forth along a 25 m walkway and were allowed to stop and rest during the test. This test measured the distance that an individual could walk on a flat, hard surface over a period of 6 min. The reliability of the 6MWT in individuals with stroke is high [32].

The Chinese version of the Fall Efficacy Scale–International (FES–I) was used to evaluate concerns about falling. The FES–I is a 16-item questionnaire that is designed to assess confidence in performing activities of daily living without falling [33]. The questionnaire asks participants how confident they are that they could perform 16 daily activities without falling on a 4-point scale, ranging from 1–4 (1 = not at all concerned, 4 = very concerned) and giving a summary score between 16 and 64. Lower scores indicate less concern of falling in maintaining daily living activities. This scale has been shown to have good validity and high reliability [33].

### 2.6. Statistical Analysis

All analyses were performed using the SPSS 24.0 statistical package (SPSS Inc., Chicago, IL, USA). The data were presented as means (standard deviation). Due to the small sample size and non-normal data distribution, nonparametric statistical procedures were employed for analysis. An intention-to-treat analysis was conducted, substituting missing values with means of group data. General characteristics and pre-intervention variables between groups were compared using the Mann–Whitney U test for continuous variables and a Chi-square test for categorical variables. Friedman’s test, followed by the Wilcoxon signed-rank test for post-hoc analysis, was used for within-group comparisons. Change values for the variables were calculated by subtracting the pre-intervention data from the post-intervention data or by subtracting the pre-intervention data from the follow-up data and analyzed using the Mann–Whitney U test for between-group comparisons. The statistical significance was set at *p* ≤ 0.05.

## 3. Results

**Figure 1:**
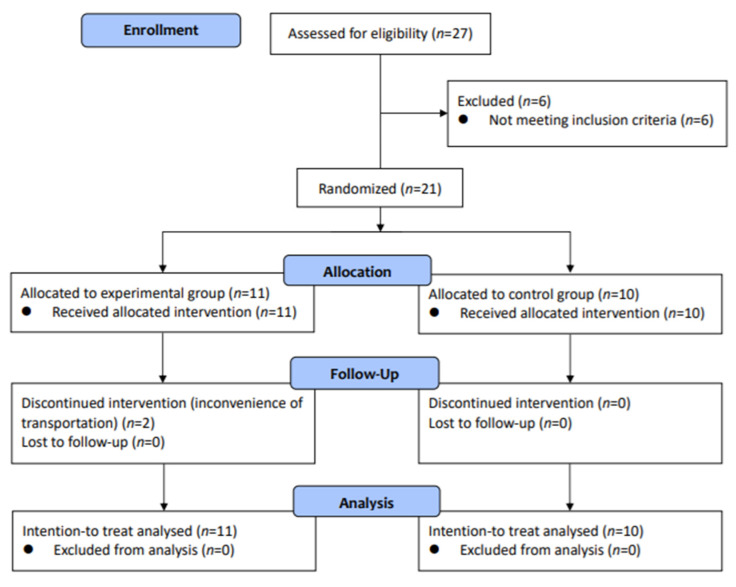
Flowchart of the experimental design.

A total of 27 potentially eligible individuals with chronic stroke were screened and 21 were recruited. Then, 11 participants were randomly allocated to the experimental group, and 10 were allocated to the control group. Of the 21 participants, 2 in the experimental group did not complete the intervention because of personal reasons. The 19 participants who completed the intervention attended all of the intervention sessions. A flow diagram of the study protocol is shown in Figure 1. None of the participants reported any adverse events.

The demographic characteristics of the participants in both groups are summarized in Table 1. The groups were similar with respect to age, gender, height, weight, affected side, time since stroke, and duration of cane use (Table 1). The differences in all pre-intervention measures between the groups were not found to be statistically significant (Table 2 and Table 3).

The results of gait performance are presented in Table 2. Within-group analysis revealed that adjunct non-elastic hip taping significantly decreased the double-support time (*p* = 0.003) and spatial symmetry index (*p* = 0.033) at post-intervention. The positive effects on the double-support time (*p* = 0.004) and spatial symmetry index (*p* = 0.021) were maintained up to the 1-month follow-up. Adjunct sham taping did not show a significant improvement in any of these gait measures. Compared with the control group, the experimental group exhibited a better improvement in the double-support time (*p* = 0.024) at post-intervention.

The results of the secondary outcomes are shown in Table 3. Within-group analysis revealed that both the experimental and control groups exhibited significant improvements in the BBS, 6MWT, and FES–I at post-intervention and 1-month follow-up. However, there was no significant difference between the two groups in the BBS, 6MWT, and FES–I results.

## 4. Discussion

This is the first randomized controlled trial to examine the effect of non-elastic hip taping combined with exercise training on gait performance in cane-assisted ambulators with chronic stroke. The results demonstrated that adjunct non-elastic hip extensor and abductor taping enhanced the effects of exercise training on gait stability and gait symmetry in cane-assisted ambulators with chronic stroke. Both non-elastic hip taping and sham taping combined with exercise training showed improvements in balance, endurance, and concern about falling after intervention. The current findings provide evidence that the application of adjunct non-elastic hip taping is promising for improving gait performance, especially in regard to stability and symmetry, post-stroke.

A cane is often prescribed to improve stability for those with impaired balance and gait post-stroke. Improvement in gait stability may help patients to discard the cane. The double-support time is around 20~22% in the normal gait cycle [34,35]. Increase in the double-support time represents gait instability [36]. The participants in the present study showed a high baseline value in double-support time at pre-intervention. Our results demonstrated that double-support time was significantly decreased after non-elastic hip taping combined with exercise training, meaning that gait stability was improved. This improvement lasted for at least 1 month. Improvement in double-support time showed significant between-group differences. Some participants in the experimental group reported that they decreased the dependence on their cane when they were the familiar environments, such as home and the hospital, after the intervention. These results support the hypothesis that non-elastic hip taping can be used during therapeutic activities to improve gait stability for cane-assisted individuals with stroke.

The positive effect of adjunct non-elastic hip taping on double-support time may result partially from an improvement in pelvic stability caused by the non-elastic tape. Excessive anterior pelvic tilt and lateral pelvic tilt have often been observed in individuals with chronic stroke [37]. Pelvic malalignment has been demonstrated to highly correlate with asymmetrical weight-bearing, and it may also influence the efficiency of functional walking [15,38]. The non-elastic hip taping used in the current study was used to try to maintain the pelvis in a relatively neutral position. Our results showed that, compared with the sham taping, the non-elastic hip taping combined with exercise training significantly decreased double-support time, representing an improvement in gait stability. Consistently, Shin et al., applied posterior pelvic tilt taping to correct pelvic position and demonstrated that taping improved gait ability in people with chronic stroke [39]. In addition, a previous study reported an increase in hemiplegic hip abductor activity after hip abductor taping [25]. Based on these results, non-elastic hip taping may enhance the effects of exercise training through improvement in pelvic alignment and the activation of the hip musculature.

Another explanation may be that taping provides an increase in cutaneous sensory input. Ploughman et al. reported that a significant improvement in double-support time may result from tactile cueing added into exercise training [40]. Tactile cues may facilitate fast conducting sensory and proprioceptive fibers to initiate a more rapid muscle response [41]. In the current study, we corrected pelvic alignment to the neutral position at the beginning of each intervention to improve the biomechanical position for efficient muscle contraction, and then applied the non-elastic taping to hip muscle groups, which contained lifting hip extensors and compressing hip abductors to provide more sensory and proprioception input. According to reports from participants in the experimental group, they could easily feel a stimulation from the non-elastic tape stretching the skin during the training, which made it feel easier to move their legs, and this may have some benefits in motor relearning.

In the present study, we observed that the spatial symmetry index was significantly decreased at post-intervention and 1-month follow-up in the experimental group. Hemiparetic individuals with shorter step lengths of the affected side than those of the non-affected side reduced advancement of the paretic leg during swing due to impaired paretic hip flexor activity in pre-swing [42]. For most of our participants in the experimental group, the step length of the affected side was shorter than that of the non-affected side, and step length of the affected side was increased after intervention. The insufficient hip flexor activity of the affected side may be a potential factor resulting in the shorter step length of the affected side, leading to spatial asymmetry. Using the non-elastic hip taping which keeps the pelvis in the neutral position may increase the hip flexor activity of the affected side in the pre-swing phase and increase the step length of the affected side, resulting in improvement in the spatial symmetry index.

Although our previous study suggested that non-elastic hip taping immediately improved balance and endurance in cane-assisted ambulators, the current results showed no significant differences in these outcomes between groups. Results from previous studies support the positive effects of exercise training on improvement in balance and endurance for individuals with stroke [13,43]. Therefore, major improvements in balance and endurance were obtained through exercise training, which was the same for both groups.

There are some limitations to this study. First, the procedure of hip taping requires participants to expose their gluteal areas, and that may be unacceptable to some individuals, especially females. Thus, the numbers of males and females were uneven in the present study. Second, hip taping may work in a clinical setting, but its use is not practical in a daily setting. Third, the evaluator was not blinded to the group due to a lack of manpower in the clinical setting. However, the evaluator followed the standard operating procedure in measurement to reduce bias. Last, pelvic alignment or muscle activity was not measured in this study. Future work could evaluate the mechanism of non-elastic taping on the hip.

## 5. Conclusions

The results of the present study suggest that non-elastic hip taping combined with exercise training improved gait stability and symmetry in cane-assisted ambulators post-stroke. The improvements in gait stability and spatial symmetry may last for at least 1 month. The findings of the current study support that non-elastic hip taping can be used as an adjunct therapy during exercise training to improve functional performance in individuals with chronic stroke.

## Figures and Tables

**Table 1:** Demographic and baseline characteristics of study participants.

Characteristics	Experimental Group (*n* = 11)	Control Group (*n* = 10)	*p*
Age (years)	62.27 ± 10.10	63.30 ± 7.05	0.58
Gender (male/female)	8/3	7/3	0.63
Height (cm)	166.32 ± 7.23	165.80 ± 6.82	0.97
Weight (kg)	67.44 ± 14.25	65.80 ± 10.06	0.86
Affected side (right/left)	5/6	5/5	0.61
Time since stroke (months)	32.55 ± 33.84	60.90 ± 61.98	0.28
Duration of cane use (months)	29.27 ± 34.36	57.00 ± 61.79	0.31

Data are presented as the mean ± standard deviation or proportion.

**Table 2:** Comparisons of gait performance.

Measures	Experimental Group (*n* = 11)			Control Group (*n* = 10)		
	Pre-Intervention	Post-Intervention	Follow-Up	Pre-Intervention	Post-Intervention	Follow-Up
Velocity (m/s)	0.45 ± 0.24	0.48 ± 0.29	0.48 ± 0.27	0.41 ± 0.25	0.40 ± 0.29	0.44 ± 0.26
Change value		0.03 ± 0.14	0.03 ± 0.11		−0.01 ± 0.12	0.04 ± 0.10
Double-support time (%)	34.71 ± 3.96	26.32 ± 5.05 *	31.29 ± 2.86 *	32.42 ± 10.05	29.96 ± 6.36	32.86 ± 9.80
Change value		−8.39 ± 3.27 ^†^	−3.41 ± 2.50		−2.45 ± 6.29	0.44 ± 7.56
Spatial symmetry index (%)	15.49 ± 9.10	9.80 ± 5.45 *	7.54 ± 7.93 *	14.10 ± 12.09	14.87 ± 6.43	11.71 ± 7.63
Change value		−5.70 ± 7.21	−7.95 ± 8.66		0.77 ± 13.34	−2.39 ± 14.48
Temporal symmetry index (%)	20.35 ± 14.13	28.26 ± 15.52	16.39 ± 8.73	24.38 ± 20.29	20.30 ± 14.37	20.45 ± 11.23
Change value		7.91 ± 12.64	−3.96 ± 15.08		−18.28 ± 48.23	−3.93 ± 20.77

Data are presented as mean ± standard deviation. * and ^†^ are *p* ≤ 0.05 for within-group (Post-intervention vs. Pre-intervention; Follow-up vs. Pre-intervention) and between-group comparisons, respectively.

**Table 3:** Comparisons of balance, endurance, and concern about falling.

Measures	Experimental Group (*n* = 11)			Control Group (*n* = 10)		
	Pre-Intervention	Post-Intervention	Follow-Up	Pre-Intervention	Post-Intervention	Follow-Up
Berg Balance Scale	42.27 ± 3.90	50.22 ± 3.34 *	48.47 ± 4.22 *	40.40 ± 5.72	48.40 ± 5.38 *	47.80 ± 5.71 *
Change value		7.95 ± 2.87	6.19 ± 2.55		8.00 ± 3.23	7.40 ± 3.06
6-min walk test (m)	178.55 ± 85.18	200.50 ± 84.88 *	199.43 ± 86.77 *	174.81 ± 89.29	201.16 ± 113.95 *	204.56 ± 118.29 *
Change value		21.95 ± 31.88	20.89 ± 33.65		26.35 ± 36.75	29.75 ± 40.94
Fall Efficacy Scale	33.91 ± 8.17	22.22 ± 3.46 *	21.89 ± 3.08 *	33.50 ± 7.58	25.00 ± 8.01 *	25.50 ± 7.21 *
Change value		−11.69 ± 6.37	−12.02 ± 6.23		−8.50 ± 5.10	−8.00 ± 6.50

Data are presented as mean ± standard deviation. * is *p* ≤ 0.05 for within-group comparison (Post-intervention vs. Pre-intervention; Follow-up vs. Pre-intervention).

## Data Availability

The data that support the findings of this study are available from the corresponding author upon reasonable request.
